# Trends and determinants of teenage childbearing in Ethiopia: evidence from the 2000 to 2016 demographic and health surveys

**DOI:** 10.1186/s13052-019-0745-4

**Published:** 2019-11-29

**Authors:** Getachew Mullu Kassa, Ayodele O. Arowojolu, Akin-Tunde Ademola Odukogbe, Alemayehu Worku Yalew

**Affiliations:** 1 Department of Obstetrics and Gynaecology, College of Medicine, Pan African University Life and Earth Sciences Institutes, University of Ibadan, Ibadan, Nigeria; 2 College of Health Sciences, Debre Markos University, P.O.BOX: 269, Debre Markos, Ethiopia; 3 Department of Obstetrics and Gynaecology, College of Medicine, University of Ibadan, University College Hospital, Ibadan, Nigeria; 4 School of Public Health, College of Health Sciences, Addis Ababa University, Addis Ababa, Ethiopia

**Keywords:** Adolescents, Demographic and health survey, Ethiopia, Teenage childbearing, Teenage pregnancy

## Abstract

**Background:**

Teenage childbearing among adolescents aged 15 to 19 is a common sexual and reproductive health (SRH) issue among young people, particularly in developing countries. It is associated with higher maternal and neonatal complications. Almost half (47%) of the population in Ethiopia are young people under 15 years old. Therefore, a clear understanding of the trend and determinants of teenage childbearing is essential to design proper intervention programs.

**Methods:**

Secondary analysis of the 2000 to 2016 Ethiopia Demographic and Health Survey (DHS) data were conducted. A total of 3710 (DHS 2000), 3266 (DHS 2005), 4009 (DHS 2011) and 3381 (DHS 2016) adolescents (aged 15 to 19 years old) were included from the four surveys. The main outcome variable of this study was teenage childbearing, and independent variables were categorized into individual- and community-level factors. The 2016 DHS was used to identify the factors associated with teenage childbearing. Multi-level logistic regression analysis technique was used to identify the factors associated with teenage childbearing. The analysis was adjusted for different individual- and community- level factors affecting teenage childbearing. Data analysis was conducted using STATA software.

**Results:**

The prevalence of adolescents who started childbearing reduced from 16.3% in 2000 DHS to 12.5% in 2016 DHS, *p-value = < 0.0001*. From the 2016 DHS, the percentage of adolescents who have had a live birth was 10.1%, and the percentage of adolescents who were currently pregnant was 2.4%. The highest percentage of teenage childbearing was in Affar region (23.4%), and the lowest was in Addis Ababa city (3%). The odds of teenage childbearing was higher among adolescents in the age range of 18–19 years old (AOR = 2.26; 95% CI: 1.29, 3.94, *p-value < 0.01),* those who started sexual intercourse before their eighteenth birthday (AOR = 12.74; 95% CI: 4.83, 33.62, *p-value < 0.001*), who were married or living together (AOR = 8.98; 95% CI: 2.49, 32.41, *p-value < 0.01),* and among those who were widowed, divorced or separated (AOR = 4.89; 95% CI: 1.36, 17.61, *p-value < 0.05*).

**Conclusions:**

One in ten teenage girls have already started childbearing in Ethiopia. Variations were observed in the percentage of teenage childbearing across different sociodemographic- and economic variables. Factors like age, early sexual initiation before 18 years of age, ever married, and geographical region were significant factors associated with teenage childbearing. School- and community- based intervention programs aimed at prevention of early marriage and early sexual intercourse is essential to reduce teenage childbearing and its complications.

## Introduction

Adolescents (people aged 10 to 19 years) are considered a critical target population which can influence the global public health [1, 2]. More than 50% of the world population are young people aged below 25 years, and nearly 85% of the world’s adolescent population live in developing countries, particularly in Sub-Saharan Africa [2]. A recent estimate of World Health Organization (WHO) in 2016 showed around 1 in 6, about 1.2 billion, people globally are adolescents [3].

Adolescents are prone to early sexual intercourse, unwanted pregnancy, abortion, HIV infection, substance abuse, child marriage and other sexual and reproductive health (SRH) problems [1]. Even though a marked reduction in teenage pregnancy has been observed since 1990, still more than 11% of all childbirths globally are to girls aged 15 to 19 years old [4, 5]. More than 95% of these childbirths occur in low and middle-income countries (LMIC) [5]. In 2018, the global rate of adolescent pregnancy was 44 births per 1000 girls aged 15 to 19 [3].

However, most adolescents have immature reproductive organs for pregnancy and childbearing and are also not psychosocially prepared [6]. As a result, pregnancy in this age group is responsible for potentially poor obstetric and neonatal outcomes [7, 8]. Pregnancy and childbirth complications are the leading causes of death among women aged 15 to 19 in LMIC [4]. Serious maternal health consequences include: pregnancy induced hypertension (PIH) [9, 10], postpartum hemorrhage (PPH) [10, 11], and anemia [10], among others. Additionally, peripartum and neonatal problems associated with teenage childbearing include: preterm labor [12–15], low birth weight (LBW) [12, 14–16], still birth [17, 18], and perinatal and neonatal mortality [19].

Ethiopia is the second most populous country in Africa and is characterized by high population growth of 2.5% annually [20]. The fertility rate was 5 children per woman (2017), and most of the population are young people. In 2016, nearly half (47%) of the total population was under 15 years old [21]. In addition, a large proportion (20%) of the population are aged 15–24 [22], of whom 47% are sexually active [21].

Adolescents in Ethiopia are exposed to several SRH problems. The median age at first sexual intercourse among women aged 25–49 was 16.6 years [21]. Sixty two percent of women have their first sexual intercourse before the age of 18, and 76% have by age 20 [21]. Problems like STIs/HIV [23], nutritional problems like stunting and underweight [24, 25], substance use [26], and mental health problems [27] are common among adolescents in Ethiopia. According to the 2016 EDHS report, the percentage of HIV positive adolescents was 0.1%. Additionally, the percentage of thin adolescents (Body Mass Index < 18.5) was 65.9 and 36.1% among men and women adolescents, respectively [21]. The magnitude of substance use like lifetime alcohol consumption (33.95%), khat chewing (24.69%) and cigarette smoking (20.38%) was also higher among young people in Ethiopia, with higher odds of substance use among males than females [28]. The problems of poor SRH outcomes among adolescents are further aggravated by poor socioeconomic status, poor access to SRH services, and harmful traditional practices like early marriage, violence, and female genital mutilation [29].

Teenage pregnancy is a major SRH problem among young people in Ethiopia. The 2000, 2005, 2010 and 2016 Ethiopia Demographic and Health Survey (EDHS) reports showed teenage pregnancy rates of 16, 17, 12 and 13%, respectively [7, 8, 21, 30]. According to the 2013 UNFPA report, Ethiopia was ranked among the top 10 countries with the highest number of women aged 20 to 24 years old and who gave birth by their eighteenth birthday [31]. Furthermore, adolescent girls and young women in Ethiopia are responsible for more than 45% of total births [29].

Teenage pregnancy is also associated with high maternal mortality ratio (MMR)- maternal deaths per 100,000 livebirths [32]. The MMR in Ethiopia was 1250 per 100,000 live births in 1990; which fell to an estimated 353 per 100,000 livebirths in 2015 [33]. Despite a good progress in reducing MMR, a large number of women are still dying from pregnancy and childbirth related complications [33]. Moreover, the Sustainable Development Goal 3 (SDG-3) aims to reduce MMR to less than 70 per 100,000 live births globally by 2030 [34].

Despite the high percentage of teenage childbearing in Ethiopia, factors associated with adolescent pregnancy are not well investigated. This study, therefore, was conducted to assess the trend and determinants of teenage childbearing using 2000 to 2016 EDHS. The findings from this study will be essential for a clear understanding of the situation and to design intervention programs to reduce teenage childbearing and related complications, and thereby improve sexual and reproductive health of adolescents.

## Methods and materials

### Study setting

According to the World Bank, Ethiopia is the second most populous country in Africa, with a population of 105 million in 2017 [35]. According to the 2016 EDHS report, almost half (47%) of Ethiopian population are young people under 15 years old [21]. Ethiopia is structured into nine regions and two city administrations. The regions include: Tigray, Affar, Amhara, Oromiya, Somali, Benishangul-Gumuz, Southern Nations Nationalities and People (SNNP), Gambela, and Harari. Administrative cities include; Addis Ababa and Dire Dawa [8, 21].

### Data source and population

This study used data from the four Ethiopia Demographic and Health Surveys- the 2000 [7], 2005 [30], 2011 [8], and 2016 [21] for the descriptive statistics and to identify the trend of teenage childbearing. For the second objective, to determine factors associated with teenage childbearing, data from the 2016 DHS were used. All surveys collected data on household characteristics, women aged 15–49, and men aged 15–59 [7, 8, 21, 30]. The current study used data from the women’s questionnaire, particularly data of adolescent women (aged 15–19) were extracted from all national surveys.

### Sample size and sampling methods

The sample for all DHS surveys were designed to represent all regions and administrative cities in the country. The survey participants were selected using stratified and two stage sampling methods: enumeration areas (EAs) in the first stage and households in the second stage. Each region was stratified into urban and rural areas. Then probability proportional allocation to sample size was made. For the 2016 DHS, 645 enumeration areas (EAs) were selected. From this, 202 EAs were from urban and 443 were from rural areas [21]. The 2011 DHS included 624 EAs (187 from urban and 437 from rural areas) [8]. The 2005 DHS included 540 EAs (145 from urban and 395 from rural areas) [30], and 539 EAs (138 from urban ad 401 from rural) were included in the 2000 DHS [7].

A representative sample of 14,072 households were successfully interviewed in the 2000 DHS (response rate 96%) [7], 13,721 households in 2005 DHS (response rate 99%) [30], 16,702 households in 2011 DHS (response rate 98%) [8], and 16,650 households (response rate 98%) were interviewed in the 2016 DHS [21]. The number of adolescents aged 15–19 included in the 2000 DHS were 3710, in 2005 DHS were 3266, in 2011 were 4009, and 3381 adolescents participated in the 2016 DHS [7, 8, 21, 30].

### Data collection instrument and period

The DHS uses three core questionnaires adapted from the MEASURE DHS project. These questionnaires include the household, women’s and men’s questionnaires [7, 8, 21, 30]. Additional questionnaires include: the biomarker questionnaire and the health facility questionnaire. This study used data from the women’s questionnaire of the surveys. The data collection tool was first prepared in English and then translated in to the three main languages in the country, Amharic, Oromiffa, and Tigrigna languages for the 2005 to 2016 DHS [8, 21, 30]. The 2000 DHS also used additional Somaligna and Afarigna languages [7]. Pretest was conducted before the data collection period, and training was provided for all data collectors, supervisors, and quality controllers involved in the field work [7, 8, 21, 30].

The 2000 DHS was conducted from February to May, 2000 [7], the 2005 DHS from April 27 to August 30, 2005 [30], and the 2011 DHS survey data collection was conducted from December 27, 2010 to June 3, 2011 [8]. The data collection period for 2016 DHS was from January 18, 2016 to June 27, 2016 [21].

### Variables of the study

#### Outcome variables

The main outcome variable of this study was teenage childbearing. It is defined as the percentage of teenagers who are mothers, pregnant with their first child, and have begun childbearing [36]. These included all women between the age of 15 to 19 years old at the time of interview. The percentage of adolescent women who are mothers was calculated by dividing the number of adolescent women who have had a birth by the total number of teenage women including those women without a birth. Percentage of women that are pregnant with first child was calculated by dividing the number of women that have not had a birth but who are pregnant at the time of data collection by the total number of teenage women including those women without a birth. The percentage of women who have begun childbearing was calculated by adding the number of women who either have had a birth or who are pregnant at the time of interview and dividing by the total number of teenage women including those women without a birth [36].

#### Independent variables

**Fig. 1:**
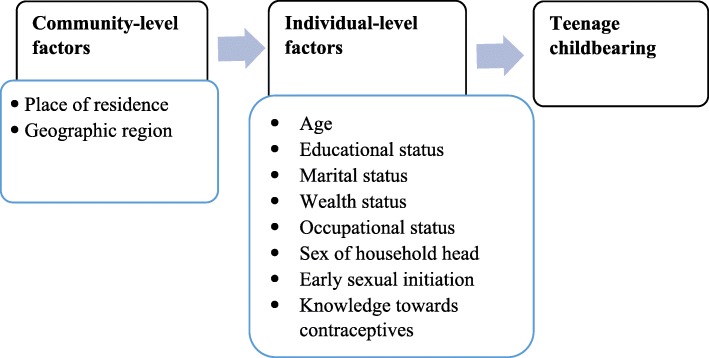
Analysis framework for factors associated with teenage childbearing

The independent variables were categorized into two level factors: individual-level, and community-level factors. The individual-level factors include: age of respondents, educational status, wealth status, occupational status, marital status, sex of household head, early sexual initiation (sexual intercourse before 18 years old), *Khat* chewing, and knowledge towards contraceptive methods. Community-level factors include: place of residence (urban vs rural) and geographic region (Fig 1).

### Data processing and analysis

After data collection, completed DHS questionnaires were carefully coded, entered, and edited [8]. Data analysis used the weighted samples to ensure the survey results were representatives of the national and regional level findings. Data analysis was conducted using STATA software (version 14; StataCorp, College Station, TX). Descriptive statistics like frequency and percentage were used. The demographic characteristics of respondents and outcome variables were compared across the four surveys. Except the 2000 DHS, all other surveys reported wealth index. To estimate the wealth index for the 2000 DHS, Principal Component Analysis was used from the household possession of items, floor and roof materials, type of toilet facility, and type of water source. The trend analysis of teenage childbearing was assessed using the Extended Mantel-Haenszel chi square test for linear trend using the OpenEpi (Version 3.01)- Dose Response program [37]. A *p-value* less than 0.05 was used to declare a 95% significant probability of existence of trend.

Multi-level logistic regression analysis technique was used to identify the factors associated with teenage childbearing. A total of four modellings were conducted. The first model was an empty model, which was conducted to estimate the random variability in the intercept. The second model was conducted to estimate the effect of individual- level factors on teenage childbearing. The third model assessed the effect of community- level factors on teenage childbearing. Finally, model four estimated the effect of both individual- and community-level factors on teenage childbearing. The Intra-Cluster Correlation (ICC) was calculated to show between-cluster correlation within a model. The Proportional Change in Variance (PCV) was also calculated to determine the power of variables included in each model in predicting teenage childbearing. The model with the highest PCV value was considered to identify the factors associated with teenage childbearing. Variables with *p-value* less than 0.05 were taken as significant factors.

### Ethical considerations and data set access

All Ethiopian Demographic and Health Surveys were conducted after obtaining ethical clearance from Ethiopia Health and Nutrition Research Institute Review Board, the Ministry of Science and Technology, Institutional Review Board of ICF International, and the CDC. The overall process of the survey, including coordination of activities, questionnaire design, training of data collectors, supervisors and all people involved in the process and report writing were strictly followed. Data were collected after taking informed consent, and all information was kept confidential [8]. For this specific research, permission was given by the Demographic and Health Surveys Program to access EDHS data after review of the submitted brief descriptions of the study to the DHS program. The datasets were treated with utmost confidentially.

## Results

### Sociodemographic characteristics

The mean ages (+standard deviations (SDs)) of adolescents included in the four surveys were similar, the latest (2016 DHS) was 16.9 + 1.34 years. About one-fourth of adolescents included in the 2016 DHS were from urban area. In addition, 17% of the adolescents were married. There was reduction in the percentage of not educated adolescents, which reduced from 61% in 2000 DHS to 13.9% during the 2016 DHS. On the other hand, the percentage of adolescents who were not currently working increased from 50.4% (2000 DHS) to 75.7% (2016 DHS) (Table 1).

**Table 1:** Sociodemographic characteristics of adolescents in Ethiopia using the 2000 to 2016 DHS

Variables		2000 DHS *n* (%)	2005 DHS *n* (%)	2011 DHS *n* (%)	2016 DHS *n* (%)
Age (years)	(mean + SD)	16.71 + 1.4	16.9 + 1.4	16.8 + 1.4	16.9 + 1.34
Residence	Urban	816 (22)	703 (21.5)	1042 (26)	805 (23.8)
	Rural	2894 (78)	2562 (78.5)	2968 (74)	2576 (76.2)
Religion	Orthodox	1881 (50.7)	1703 (52.2)	2023 (50.5)	1426 (42.2)
	Catholic	62 (1.7)	45 (1.4)	36 (0.9)	22 (0.7)
	Protestant	545 (14.7)	606 (18.6)	833 (20.8)	847 (25)
	Muslim	1098 (29.6)	859 (26.3)	1075 (26.8)	1064 (31.5)
	Traditional	116 (3.1)	28 (0.9)	20 (0.5)	16 (0.5)
	Other	6 (0.2)	24 (0.7)	17 (0.4)	6 (0.2)
Marital status	Never married	2597 (70.0)	2394 (73.3)	3087 (77)	2642 (78.1)
	Married	851 (22.9)	689 (21.1)	707 (17.6)	569 (16.8)
	Living together	11 (0.3)	21 (0.7)	58 (1.5)	19 (0.6)
	Widowed	8 (0.2)	8 (0.2)	4 (0.1)	1 (.0)
	Divorced	190 (5.1)	132 (4.0)	127 (3.2)	98 (2.9)
	No longer living together/separated	53 (1.4)	20 (0.6)	27 (0.7)	53 (1.6)
Educational status	No education	2265 (61.0)	1308 (40.1)	695 (17.3)	469 (13.9)
	Primary	977 (26.3)	1423 (43.6)	2813 (70.2)	2148 (63.5)
	Secondary	468 (12.6)	519 (15.9)	406 (10.1)	678 (20)
	Higher	-	16 (0.5)	95 (2.4)	87 (2.6)
Currently working	No	1872 (50.4)	2494 (76.4)	2920 (72.8)	2558 (75.7)
	Yes	1836 (49.5)	770 (23.6)	1086 (27.1)	823 (24.3)
Wealth Quantile	Lowest	472 (12.7)	448 (13.7)	686 (17.1)	478 (14.1)
	Second	1915 (51.6)	566 (17.3)	696 (17.4)	558 (16.5)
	Middle	190 (5.1)	627 (19.2)	687 (17.1)	638 (18.9)
	Fourth	534 (14.4)	603 (18.5)	889 (22.2)	716 (21.2)
	Highest	600 (16.2)	1022 (31.3)	1051 (26.2)	992 (29.3)
Total		3710 (100)	3266 (100)	4009 (100)	3381 (100)

### Sexual and reproductive history of adolescents

The mean (+ SD) age at first marriage or cohabitation was 14.34 + 2.35 during the 2000 DHS, which increased to 15.5 + 1.7 during the 2016 DHS. Age at first birth was 16.5 + 1.4 during 2016 DHS. Almost one-fifth (18.3%) of adolescents started sexual intercourse before the age of 15 during the 2016 DHS. The percentage of adolescents who started sexual intercourse before 18 years old reduced from 27% during the 2000 DHS to 21.9% in the 2016 DHS. Similarly, the percentage of adolescents who were married or started cohabiting before their eighteenth birthday reduced from 27.2% (2000 DHS) to 19.2% (2016 DHS).

The percentage of adolescents who knew no contraceptive methods reduced from 31.2% (2000 DHS) to 3% (2016 DHS). Similarly, the percentage of adolescents who knew modern methods increased from 68.4 to 96.9%. Only 1.3% of adolescents were using modern contraceptive methods during the 2000 DHS, which increased to 7.5% during the 2016 DHS, while the percentage of adolescents who didn’t intend to use contraceptive methods reduced from 47.1% (2000 DHS) to 25.3% (2016 DHS) (Table 2).

**Table 2:** Sexual and reproductive health history of adolescents in Ethiopia using the 2000 to 2016 DHS

Variables		2000 DHS *n* (%)	2005 DHS *n* (%)	2011 DHS *n* (%)	2016 DHS *n* (%)
Age at first birth (in years)	(mean + SD)	16.44 + 1.44	16.2 + 1.5	16.4 + 1.44	16.5 + 1.4
Age at first marriage/ cohabitation (in years)	(mean + SD)	14.34 + 2.35	14.34 + 2.4	15.1 + 2.12	15.5 + 1.7
Had sexual intercourse before age 15	No	623 (16.8)	525 (16.1)	666 (16.6)	620 (18.3)
	Yes	499 (13.5)	363 (11.1)	285 (7.1)	212 (6.3)
	Never had sex	2571 (69.3)	2362 (72.3)	3038 (75.8)	2549 (75.4)
Early sexual intercourse (before 18 years old)	No	121 (3.3)	82 (2.5)	136 (3.4)	91 (2.7)
	Yes	1002 (27)	806 (24.7)	815 (20.3)	741 (21.9)
	Never had sex	2571 (69.3)	2362 (72.3)	3038 (75.8)	2549 (75.4)
Age at first marriage/cohabitation (before 18 years old)	No	105 (2.8)	69 (2.1)	126 (3.1)	89 (2.6)
	Yes	1008 (27.2)	802 (24.6)	796 (19.9)	650 (19.2)
	Never cohabited	2597 (70)	2394 (73.3)	3087 (77.0)	2642 (78.1)
Gave birth before age 15	No	434 (11.7)	386 (11.8)	363 (9.1)	321 (9.5)
	Yes	41 (475)	57 (1.7)	41 (1.0)	20 (0.6)
	Never gave birth	3235 (87.2)	2822 (86.4)	3605 (89.9)	3041 (89.9)
Knowledge of any contraceptive methods	Knows no method	1157 (31.2)	616 (18.9)	176 (4.4)	101 (3.0)
	Knows only traditional method	16 (0.4)	0	3 (0.1)	4 (0.1)
	Knows modern method	2537 (68.4)	2649 (81.1)	3830 (95.5)	3276 (96.9)
Ever terminated pregnancy	No	3640 (98.1)	3243 (99.3)	3973 (99.1)	3350 (99.1)
	Yes	70 (1.9)	23 (0.7)	36 (0.9)	31 (0.9)
Fertility preference	Have another pregnancy	2739 (73.8)	2303 (70.5)	3326 (82.9)	2503 (74.0)
	Undecided	524 (14.1)	170 (5.2)	207 ()5.2	632 (18.7)
	No more	436 (11.8)	716 (21.9)	471 (11.7)	245 (7.3)
	Declared infecund	10 (0.3)	64 (2.0)	2 (.0)	1 (.0)
Contraceptive use and intention	Using modern method	47 (1.3)	80 (2.5)	208 (5.2)	251 (7.4)
	Using traditional method	8 (0.2)	3 (0.1)	6 (0.1)	3 (0.1)
	Non-user-intends to use later	1909 (51.5)	2007 (61.4)	2900 (72.3)	2270 (67.1)
	Does not intend to use	1746 (47.1)	1176 (36.0)	895 (22.3)	857 (25.3)

### Magnitude of teenage childbearing

The percentage of teenagers already childbearing increased with age, 1.2% for adolescents aged 15 and 39.7% for those aged 19 years old in 2000 DHS, and from 1% for those aged 15 to 33.6% for those aged 19 years old in 2011 DHS. In the first three surveys, the prevalence of teenage childbearing was highest in Gambela region, with 26% (in 2000), 30.8% (in 2005), and 20.5% (in 2011). In 2016, the prevalence of teenage childbearing was highest (23.4%) in Affar region. In all of the four surveys, the prevalence of teenage childbearing was higher among rural adolescents than those from the urban areas. For those from the urban areas, the prevalence of teenage childbearing reduced from 9.1% in 2000 DHS to 4.9% in 2016 DHS. Similarly, it reduced from 18.3 to 12.6% for adolescents from rural areas (Table 3).

**Table 3:** Magnitude of teenage childbearing by background characteristics in Ethiopia, findings from 2000 to 2016 DHS data

Variables	Percentage of teenagers who started childbearing							
	2000 DHS		2005 DHS		2011 DHS		2016 DHS	
	%	95% CI	%	95% CI	%	95% CI	%	95% CI
Region								
Tigray	20.9	17, 25.6	14.7	11.2, 19	12	8.5, 16.5	12.0	8.6, 16.3
Affar	21.1	16, 27.2	20.3	14, 28.7	4.6	3.2, 6	23.4	17.3, 30.8
Amhara	25	20.4, 30.3	20.3	16.6, 24.7	11.6	8.6, 15.3	8.3	5.6, 12.2
Oromiya	15.8	12.7, 19.5	19	15.5, 23	15.8	12.1, 20.4	17.0	13.0, 21.9
Somalia	12.7	8.1, 19.4	19.5	11.1, 32	19.2	12.8, 27.8	18.7	13.3, 25.6
Benishangul-Gumuz	22.2	13, 35.3	27.1	18.6, 37.7	19.3	14.8, 24.8	13.6	9.2, 19.6
SNNPR	8.1	5.8, 11.1	11	7.6, 15.6	8.2	5.9, 11.2	10.7	7.8, 14.4
Gambela	26	14.6, 42	30.8	20.9, 42.8	20.5	13.7, 29.5	16.2	11.9, 21.8
Harari	12.9	9.1, 17.9	21.9	17.1, 27.6	14.5	10, 20.5	16.9	11.4, 24.4
Addis Ababa	4.7	3.3, 6.8	4.3	2.6, 6.8	2.6	1.1, 6.1	3.0	1.6, 5.6
Dire Dawa	11	6.5, 17.9	13.7	10.6, 17.5	7.6	4.4, 13	12.5	8.1, 18.8
Place of residence								
Urban	9.1	6.2, 13.3	6.6	4.6, 9.5	4.1	2.6, 6.5	4.9	3.2, 7.5
Rural	18.3	16.3, 20.4	19.4	17.1, 21.9	15.3	13, 17.8	14.8	12.6, 17.4
Highest educational level								
No education	20.8	18.6, 23.3	28.9	25.3, 32.9	32.8	27.8, 38.2	27.9	22.1, 34.5
Primary	8.9	6.6, 11.9	10.4	8.2, 13.2	8.8	7.2, 10.7	12.1	10.0, 14.6
Secondary	9.5	6.3, 14.2	3.1	1.8, 5.2	21.1	6.2, 36	4.1	2.6, 6.4
Higher	-	-	-	-	-	-	3.4	0.6, 16.4
Religion								
Orthodox	18.4	16, 21.2	15.9	13.5, 18.5	11.4	9, 14.2	7.7	5.9, 9.8
Catholic	7.8	1.7, 29.3	7	1.9, 22.5	8.1	2.4, 23.6	0.4	0.1, 3.7
Protestant	11.8	8.7, 15.8	11.1	7.5, 16.1	11.7	8.1, 16.8	10.4	7.4, 14.3
Muslim	15.6	12.6, 19.2	22.9	18.7, 27.7	14.5	11.2, 18.7	20.5	16.6, 25.0
Traditional	13.2	5.5, 28.4	0.8	0.2, 2.8	29	9.8, 60.5	31.4	27.5, 35.7
Other	-	-	23.3	7, 55.3	2.4	0.5, 11.2	28.3	9.0, 61.2
Total	16.3	14.4, 18.3	16.6	14.8, 18.7	12.4	10.6, 14.4	12.5	10.6, 14.6

### Trends of teenage childbearing

**Fig. 2:**
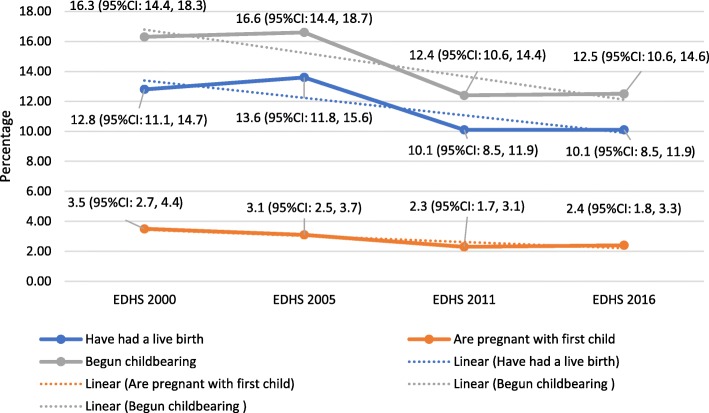
Trend of teenage childbearing in Ethiopia using data from 2000 to 2016 DHS

The prevalence of adolescents who started childbearing reduced from 16.3% (95% CI, 14.4, 18.3) in 2000 DHS to 12.5% (95% CI, 10.6, 14.6) in 2016 DHS. Similarly, the percentage of adolescents who have had a live birth reduced from 12.8% (95% CI, 11.2, 14.6) to 10.1% (95% CI, 8.5, 11.9) between 2000 and 2016 DHS, respectively. The percentage of adolescents who are currently pregnant also reduced from 3.5% (95% CI, 2.7, 4.4) to 2.4% (95% CI, 1.8, 3.3) during the 2000 and 2016 DHS, respectively. Moreover, the reduction was statistically significant for teenage childbearing (*p-value < 0.0001*), adolescents who have had a live birth (*p-value < 0.0001*), and who are pregnant with their first child (*p-value = 0.001*) outcomes from 2000 DHS to 2016 DHS (Fig. 2).

### Factors associated with teenage childbearing

Multilevel logistic regression analysis was conducted using the recent (2016) DHS data, to identify individual and community-level factors associated with teenage childbearing. The ICC in Model I (empty model) showed 8.7% variability in the teenage childbearing attributed to differences in the communities (between-cluster variability). Similarly, the between-cluster variability was 12.44% in Model II, 15.84% in Model III, and 1.19% in Model IV. According to the findings of the PCV, the addition of predictor variables to the empty model better explained the factors associated with teenage childbearing. The PCV result for Model II (individual- level factors) was 70.5%, for Model III (community-level factors) was 39.3%, and for Model IV (combined individual-and community-level factors) was 71.5%. Model IV suggests that almost 72% of the variations in the teenage childbearing between communities is explained by the individual- and community- level factors, and therefore, it better identifies the factors associated with teenage childbearing (Table 4).

**Table 4:** Results of multilevel logistic regression analysis of factors associated with teenage childbearing in Ethiopia using data from the 2016 DHS

Parameters	Model I	Model II	Model III	Model IV
Age (in years)				
15 – 17 (middle adolescence)		1		1
18 – 19 (late adolescence)		2.01 (1.2, 3.37) **		2.26 (1.29, 3.94) **
Educational status				
No education		1		1
Primary		0.86 (0.44, 1.67)		0.75 (0.37, 1.53)
Secondary		0.49 (0.16, 1.57)		0.48 (0.14, 1.66)
Higher		0.36 (0.02, 6.05)		0.57 (0.09, 3.26)
Marital status				
Never in union		1		1
Married/living together		9.09 (2.62, 31.48) **		8.98 (2.49, 32.41) **
Widowed/divorced/separated		3.17 (0.85, 11.73)		4.89 (1.36, 17.61) *
Wealth index				
Poorest		1		1
Poorer		0.66 (0.28, 1.54)		0.79 (0.31, 2.0)
Middle		0.86 (0.41, 1.8)		1.18 (0.58, 2.37)
Richer		0.81 (0.35, 1.91)		0.99 (0.42, 2.34)
Richest		0.59 (0.25, 1.39)		0.81 (0.32, 2.05)
Early sexual intercourse				
No		1		1
Yes		10.23 (3.79, 27.61) ***		12.74 (4.83, 33.62) ***
Knowledge of any contraceptive methods				
No		1		1
Yes		1.18 (0.43, 3.29)		1.55 (0.51, 4.71)
Sex of household head				
Male		1		1
Female		1.26 (0.65, 2.45)		1.23 (0.63, 2.39)
Respondent currently working				
No		1		1
Yes		1.13 (0.65, 1.94)		1.16 (0.65, 2.09)
Place of residence				
Urban			1	1
Rural			3.26 (1.75, 6.07) ***	1.56 (0.59, 4.06)
Region				
Tigray			1	1
Affar			2.39 (1.37, 4.14) **	0.75 (0.36, 1.59))
Amhara			0.62 (0.34, 1.13)	0.37 (0.18, 0.77) **
Oromiya			1.49 (0.89, 2.48)	2.62 (1.27, 5.42) **
Somalia			1.86 (1.01, 3.42) *	2.28 (1.02, 5.09) *
Benishangul-Gumuz			1.13 (0.61, 2.11)	1.19 (0.56, 2.54)
SNNPR			0.83 (0.47, 1.44)	3.08 (1.37, 6.91) **
Gambela			1.94 (1.09, 3.42) *	1.34 (0.56, 3.23)
Harari			2.09 (1.09, 4.04) *	2.27 (0.99, 5.18)
Addis Ababa			0.58 (0.23, 1.49)	1.4 (0.42, 4.66)
Dire Dawa			1.94 (0.91, 4.09)	2.46 (0.93, 6.49)
Random effect				
Community variance (SE)	0.875 (0.253)	0.258 (0.269)	0.531 (0.209)	0.249 (0.389)
ICC (%)	8.7	12.44	15.84	1.19
PCV (%)	Reference	70.5	39.3	71.5
Model fitness				
Log likelihood	-1298.88	-517.755	-1240.473	-483.652
AIC	2601.76	1067.51	2506.946	1021.304
BIC	2614.08	1144.437	2587.025	1151.119

*p <0.05, ** *p*< 0.01, *** *p*< 0.001. *AIC* Akaike's Information Criterion, *BIC* Bayesian Information Criterion, *ICC* Intra-Cluster Correlation, *PCV* Proportional Change in Variance, *SE* Standard Error

Therefore, according to the findings of Model IV, variables like age, marital status, early sexual initiation, and geographic region were significantly associated with teenage childbearing. The odds of childbearing among teenagers who were 18–19 years was about two times higher than among 15–17 years (AOR = 2.26; 95% CI: 1.29, 3.94, *p-value < 0.01).* The odds of childbearing among teenagers who were married or living together with their partner was about nine times higher (AOR = 8.98; 95% CI: 2.49, 32.41, *p-value < 0.01)*, and those who were widowed/divorced or separated was about five times (AOR = 4.89; 95% CI: 1.36, 17.61, *p-value < 0.05*) higher than adolescents who were never in union.

Additionally, the odds of teenage childbearing among teenagers who started sexual intercourse before their eighteenth birthday were about 12 times higher than those who didn’t start early sexual intercourse (AOR = 12.74; 95% CI: 4.83, 33.62, *p-value < 0.001*). Variation was also observed in the percentage of teenage childbearing across the different regions in the country. The odds of childbearing among teenagers living in Amhara region were about 63% times lower (AOR = 0.37; 95% CI: 0.18, 0.77, *p-value < 0.01*), and those living in Oromiya (AOR = 2.62; 95% CI: 1.27, 5.42, *p-value < 0.01*), Somalia (AOR = 2.28; 95% CI: 1.02, 5.09, *p-value < 0.05,* and SNNPR (AOR = 3.08; 95% CI: 1.37, 6.91, *p-value < 0.01*) were at higher odds to start childbearing than adolescents living in Tigray region (Table 4).

## Discussion

This study was conducted to assess the trend and determinants of teenage childbearing in Ethiopia using the four DHS surveys from 2000 to 2016. There was a significant reduction in the percentage of teenage childbearing from the 2000 DHS to 2016 DHS. Factors like late adolescence age, marital status, early sexual initiation, and geographical region were significantly associated with teenage childbearing.

The magnitude of teenage childbearing varied by sociodemographic characteristics. Across the three DHS (2000 to 2011), the highest prevalence of teenage childbearing was observed in Gambela region and lowest was observed in Addis Ababa, the capital city of Ethiopia. On the other hand, from the 2016 DHS, the highest percentage of teenage childbearing was in Affar region (23.4%) and the lowest was in Addis Ababa (3%). Such variations in the teenage childbearing could be attributed to the differences in the socio-demographic characteristics of teenagers, access to sexual and reproductive health services, early marriage, and knowledge towards contraceptive methods among the different geographical regions of the country. Similarly, the prevalence of teenage childbearing was also higher among rural than urban residents, and among non-educated than educated adolescents. Similar finding was also reported in a previous study conducted in Ethiopia [38]. Therefore, investment in education of teenage girls and programs aimed at increasing access towards contraceptive information and services especially for adolescents in the rural areas and regions with high prevalence of teenage childbearing is essential [39].

The prevalence of teenage childbearing reduced from 16.3% in 2000 to 12.5% in 2016 DHS. There was a slow (4%) reduction of teenage childbearing over 16 years. The finding of the current study is relatively lower compared to a systematic review and meta-analysis study conducted in Africa. The review showed that the prevalence of adolescent pregnancy in sub-Saharan African countries was 19.3% [40]. The reduction in teenage childbearing could be attributed to the increasing contraceptive use and improved access to adolescent sexual and reproductive health services in Ethiopia [7, 8, 30]. The percentage of adolescents who are using modern contraceptive methods increased from 1.3% in 2000 DHS to 7.4% in 2016 DHS. Similarly, the percentage of adolescents who are non-users and intend to use contraceptive methods increased from 51.5% in 2000 DHS to 67.1% in 2016 DHS. Moreover, contraception information and service provision is effective in reducing unintended teenage pregnancy and preventing sexually transmitted infections among young people [41]. Additionally, the reduction in the level of adolescent childbearing could also be the result of multifactorial indirect interventions like: increased urbanization, globalization, social media, and increased educational attainments.

The Ethiopian adolescent and youth health strategy (2016–2020) planned to reduce unmet need for modern contraception among adolescents from 32.8% in 2015 to 10% in 2020 [42]. The strategy indicated the mechanisms to reduce adolescent sexual and reproductive health problems. However, the poor quality of adolescent sexual and reproductive health service, inadequate infrastructures, health worker incompetence and low service utilization among adolescents were the main challenges [42]. The use of mobile health (m-health) technology to improve access and knowledge towards sexual and reproductive health information and improve SRH behavior of adolescents can also help the country reduce adolescent pregnancy and other sexual and reproductive health problems [43]. Moreover, previous study have shown the effectiveness of mHealth in improving SRH knowledge among adolescents [44].

The 2016 EDHS estimate of teenage childbearing (12.5%) is lower than other sub-Saharan African regions. The 2013 UNFPA report showed that 25% of women aged 20 to 24 years in Eastern and Southern Africa gave birth before 18 years old, and it was 28% for West and Central Africa [31]. This could be due to the high number of adolescent populations in Ethiopia compared to other sub-Saharan African countries. However, Ethiopia was ranked among the top 10 countries with the highest number of women aged 20 to 24 years old and who gave birth by their eighteenth birthday in 2013 [31]. The variations in the percentage of teenage childbearing across the different regions in Africa could be due to the differences in the study period, sociodemographic, economic circumstances, and access to adolescent SRH services between the current study area and other African regions. Moreover, the percentage of adolescent pregnancy in sub-Saharan Africa will rise to 23% by the end of 2030 [31], and this will require preventive intervention programs, particularly in Ethiopia where a large number of adolescent population are available [21].

The prevalence of teenage childbearing reduced from 16.3% in 2000 DHS to 12.5% in 2016 DHS. There was statistically significant reduction in the percentage of teenage pregnancy, teenagers who had a live birth or percentage of teenage childbearing. Therefore, intervention programs to improve access to adolescent sexual and reproductive health services, particularly in the rural areas, is essential to reduce teenage childbearing. Because, teenage childbearing is more common in the rural areas and access to sexual and reproductive health education and pregnancy prevention methods is very limited.

This study also found an increasing odd of teenage childbearing with increasing age. The percentage of teenage childbearing among adolescents aged 15 years old was 1.6% compared to 27.7% among those aged 19 years old. Similarly, adolescents in the age range of 18–19 years were more likely to start childbearing than those in the age range of 15–17 years. This could be because of higher exposure to sexual intercourse and marriage with increasing age that may result in higher childbearing [38]. Previous studies conducted in Ethiopia [45] and Kenya [46] also showed increasing odds of adolescent pregnancy with increasing age of adolescents.

The mean age at first marriage or cohabitation among teenagers in Ethiopia was relatively low, 15.5 years using the 2016 DHS. Almost one-fifth of adolescents were married or cohabited before 18 years old. Teenagers who were married or living together with a partner were almost nine times, and those who are widowed/divorced/separated were almost five times more likely to start childbearing than those who were never in a union. This could be because of the early sexual debut related with early marriage and increased encounter of sexual intercourse. Additionally, there is high unmet need for family planning (20.5%) and low level of modern method of contraceptive use (7.4%) among adolescent girls [21]. This is also because society permits initiation of intercourse once there is a union, irrespective of the age of the girl. Adolescent women who are married are exposed to early sexual intercourse and this may result in pregnancy. A study conducted in Nigeria [47] also showed significantly higher odds of adolescent pregnancy among those who were married.

However, early marriage is a violation of the rights of the adolescent girl [48]. Even though Ethiopia has ratified a Family Code and prohibited child marriage before 18 years of age [49], early marriage is a common practice across the different regions of the country. Girls who are exposed to early marriage are more likely to experience several sexual and reproductive health problems like gender-based violence, fistula, and STIs/HIV [48, 50]. Early marriage also has socioeconomic impacts like school dropout, lower future job opportunities, and lower income resulting in economic insecurity and poverty [48, 51]. A recent World Bank report showed that Ethiopia may spend billions of dollars because of child marriage [52]. Therefore, prevention of early marriage improves not only the health of women and children, but also helps to mitigate poverty and improves socioeconomic status of a country. Investment in reduction of early marriage can help to prevent early childbearing, as four out of five childbirths before 18 years old are as a result of child marriage [52]. Programs which can help reduce early marriage are important. Particularly, the use of school- and community-based intervention programs to change the cultural attitudes towards early marriage is essential [51].

This study also found a higher percentage of early sexual intercourse among teenagers. According to the 2016 EDHS data, more than one-fifth (21.9%) of teenage girls in Ethiopia started sexual intercourse before 18 years of age. Among teenage girls who already started sexual intercourse, 89% started before 18 years of age. Additionally, teenagers who started sexual intercourse before 18 years of age were more than 12 times more likely to start childbearing than those who started sexual intercourse after 18 years of age. Moreover, early sexual intercourse is a predictor of poor sexual and reproductive health outcomes like STIs/HIV among adolescents [53]. Early sexual debut was also found to be a significant determinant of teenage pregnancy in a study conducted in Baltimore and Johannesburg [54].

Previous studies have shown that religiosity is one of the factors to prevent early sexual debut. Therefore, involving religious leaders in the prevention of early sexual debut is an essential area of intervention to reduce sexual and reproductive health problems among adolescents. Moreover, intervention programs aimed at delaying the age at first sexual debut are essential. Particularly, school-based sexual and reproductive health education programs aimed at delaying sex and improving use of condom and other contraceptives are effective in preventing unintended pregnancy and other SRH complications among adolescents.

## Conclusion

One in ten adolescent girls aged 15 to 19 in Ethiopia have already started childbearing. The percentage of teenage childbearing was high despite a reduction in the recent years. There was variation in the percentage of teenage childbearing by geographic region, the highest in Affar region and lowest in Addis Ababa city. Late adolescence age, teenagers who were married or living together with a partner, and those who started sexual intercourse before 18 years old are more likely to start childbearing. Intervention programs aimed at improving knowledge and utilization of contraceptives and prevention of early sexual initiation among adolescents is essential to reduce teenage childbearing and related complications.

## Data Availability

All data pertaining to this study are contained and presented in this document.

## Abbreviations

AOR: Adjusted Odds Ratio
CI: Confidence Interval
DHS: Demographic and Health Survey
EDHS: Ethiopia Demographic and Health Survey
HIV: Human Immunodeficiency Virus
SD: Standard Deviation
SRH: Sexual and Reproductive Health
STI: Sexually Transmitted Infection
WHO: World Health Organization
